# Characterization of the Complete Nuclear Ribosomal DNA Sequences of *Paramphistomum cervi*

**DOI:** 10.1155/2014/751907

**Published:** 2014-07-20

**Authors:** Xu Zheng, Qiao-Cheng Chang, Yan Zhang, Si-Qin Tian, Yan Lou, Hong Duan, Dong-Hui Guo, Chun-Ren Wang, Xing-Quan Zhu

**Affiliations:** ^1^College of Animal Science and Veterinary Medicine, Heilongjiang Bayi Agricultural University, Daqing, Heilongjiang 163319, China; ^2^State Key Laboratory of Veterinary Etiological Biology, Key Laboratory of Veterinary Parasitology of Gansu Province, Lanzhou Veterinary Research Institute, Chinese Academy of Agricultural Sciences, Lanzhou, Gansu 730046, China

## Abstract

Sequences of the complete nuclear ribosomal DNA (rDNA) gene from five individual *Paramphistomum cervi* were determined for the first time. The five complete rDNA sequences, which included the 18S rDNA, the internal transcribed spacer 1 (ITS1), the 5.8S rDNA, the internal transcribed spacer 2 (ITS2), the 28S rDNA, and the intergenic spacer (IGS) regions, had a length range of 8,493–10,221 bp. The lengths of the investigated 18S, ITS1, 5.8S, ITS2, and 28S rDNA sequences, which were 1,994 bp, 1,293 bp, 157 bp, 286 bp, and 4,186 bp, respectively, did not vary. However, the IGS rDNA sequences had a length range of 577–2,305 bp. The 5.8S and ITS-2 rDNA sequences had 100% identity among the five investigated samples, while the identities among the IGS had a range of 53.7–99.8%. A comparative analysis revealed that different types and numbers of repeats were found within each ITS1 and IGS region, which may be related to the length polymorphism of IGS. The phylogenetic position of *P. cervi* in Paramphistomatidae was analyzed based on the 18S rDNA sequences. These results will aid in studying the intra- and interspecific variation of the Paramphistomatidae and the systematics and phylogenetics of Digenea.

## 1. Introduction

*Paramphistomum cervi* (Trematoda: Digenea: Paramphistomatidae), the representative species of the genus* Paramphistomum*, has adult flukes that customarily inhabit the rumen and immature worms that parasitize the gallbladder and reticulum of ruminants, including cattle, sheep, goat, and some wild mammals [1, 2]. Although the adult* P. cervi* is relatively less pathogenic, acute gastroenteritis can occur in young animals when several immature worms migrate through the intestine to the rumen [3–5].* P. cervi* is distributed worldwide and has been reported in many countries [1–6]. In China, Heilongjiang Province is the main endemic region [7].

Previous studies on* P. cervi* have mainly focused on morphology, life history, and epidemiology [1–7]. There are only a few molecular level studies on* P. cervi*. Recently, the complete mitochondrial DNA sequence and the ITS2 rDNA sequence of* P. cervi* were determined [8, 9]. The nuclear ribosomal DNAs (rDNAs) of eukaryotes are arranged into tandem repeats. Each repeat has a transcriptional unit containing three genes (18S, 5.8S, and 28S rRNA) with two internal transcribed spacers (ITS1 and ITS2) separating these genes and an intergenic spacer (IGS) between the transcriptional units [10]. Different rDNA regions evolved at different rates; therefore, they can be used as genetic markers for phylogenetic studies at different taxonomic levels. The ITS rDNA sequences provide useful genetic markers for parasite identification [11–14]. IGS rDNA contains some repeat sequences that cause considerable amounts of intra- and interspecific variation in parasites [15]. However, the IGS region of parasites is relatively poorly characterized.

To identify novel genetic markers for studying intra- and interspecific variation in the Paramphistomatidae and to further study the systematics and phylogenetics of Digenea trematodes, the present study determined and characterized the complete rDNA sequence of* P. cervi*, studied the intraspecific variation, and reconstructed the phylogenetic relationship of* P. cervi* within the family Paramphistomatidae.

## 2. Materials and Methods

### 2.1. Parasites and DNA Extraction

Adult* P. cervi* flukes were collected from the rumen of naturally infected cattle in Qiqihaer, Heilongjiang Province, China. Five adult flukes were washed extensively with physiological saline and identified to the species level based on morphological features described previously [2]. Total genomic DNA was extracted from five individual adult samples using the TIANamp Genomic DNA Kit (TIANGEN, Beijing, China) according to the manufacturer's instructions and eluted into 50 *μ*L double-distilled water. The obtained DNA samples were stored at −20°C until use.

### 2.2. Amplification, Sequencing, and Assembling of Complete rDNA Sequences

Six pairs of primers were designed based on the multiple alignments of* Carmyerius spatiosus* (JX518972, JX518958),* Fischoederius elongatus* (JX518979, JX518966),* Gastrothylax crumenifer* (JX518984, JX518969),* Schistosoma haematobium* (AY157263),* S. japonicum* (AY157607), and* S. mansoni* (AY157173) rDNA sequences available in GenBank. The primer sequences are listed in Table 1.

PCR reactions of 25 *μ*L contained 1 *μ*L DNA template, 5 *μ*L of 5× colorless Go* Taq* flexi buffer (pH 8.5), 2 *μ*L of MgCl_2_ (25 mM), 2 *μ*L of dNTP Mixture (2.5 mM), 0.5 *μ*L of each primer (10 pmol/*μ*L), and 0.2 *μ*L of Go* Taq* DNA polymerase (5 U/*μ*L). The reactions were performed in a thermocycler under the following conditions: 95°C for 2 min (initial denaturation), followed by 35 cycles of 95°C, 1 min (denaturation), 51.7–57.1°C for 1 min (annealing), 72°C (~1 kb region) for 1 min (extension), and a final extension of 72°C for 5 min. Each amplicon was examined in a 1.0% (w/v) agarose gel, stained with ethidium bromide (EB), and photographed upon transillumination. The DL2000 marker was used to estimate the sizes of the rDNA amplicons. Representative PCR products were sent to Life Technology Company (Beijing, China) for sequencing using the same primers used in the primary amplifications. The five complete rDNA sequences of* P. cervi* were assembled using DNAStar software.

### 2.3. Sequence Analyses and Reconstruction of Phylogenetic Relationships

The 5′ and 3′ ends of the 18S, ITS1, 5.8S, ITS2, 28S, and IGS rDNA sequences of* P. cervi* were initially determined by comparing them with previously published rDNA sequences of other trematodes. For example, the 5′ end of 18S and the 3′ end of 28S were established by a comparison with the* S. japonicum* sequence [15], the 5′ termini of ITS1 and 5.8S were established by a comparison with* Paragonimus kellicotti* (HQ900670) and* Echinostoma revolutum* (GQ463130), respectively, and the 5′ termini of ITS2 and 28S were determined by referring to a previous study on* P. cervi* isolated from Slovakia (HM026462) [9]. Sequences of these rDNA regions from different individual adult fluke were aligned separately using Clustal X 1.83 [16]. The intraspecies sequence variation in each of these rDNA regions among the five individual adults and the interspecies sequence differences of the ITS2 rDNA within the family Paramphistomatidae were determined using the MegAlign procedure in DNASTAR 5.0 [17]. The base composition, transitions, and transversions were calculated using Mega 4.0 [18]. The characteristics of the ITS1 and IGS rDNA of* P. cervi* were examined using the palindrome program in EMBOSS 6.3.1 [19] (http://mobyle.pasteur.fr/cgi-bin/portal.py?#forms::palindrome) to find inverted repeats and REPFIND [20] (http://cagt.bu.edu/page/REPFIND_submit) to identify direct repeats. These repeats were determined using the criteria of a nuclear match ≥10 bp and a mismatch ≤1.

The phylogenetic relationship of* P. cervi* with other trematodes was reconstructed based on 18S rDNA sequences, using* Taenia solium* (GQ260091) as the outgroup. A maximum parsimony (MP) analysis was performed using the PAUP 4.0 Beta 10 program [21], and 1,000 random additional searches using tree bisection-reconnection branch swapping were performed for each MP analysis. Bootstrap probability was calculated from 1,000 bootstrap replicates with 10 random additions per replicate in PAUP. A maximum likelihood (ML) analysis was performed using PUZZLE 4.1 [22]. A Bayesian inference (BI) was performed using MrBayes 3.1 [23] with four independent Markov chains run for 1,000,000 metropolis-coupled Markov chain Monte Carlo generations and sampling a tree every 1,000 generations. The consensus tree was obtained after a bootstrap analysis with 1,000 replications with values above 50% being reported. Phylograms were drawn using the Tree View program version 1.65 [24].

## 3. Results and Discussion

### 3.1. Complete rDNA Sequences

**Figure 1:**
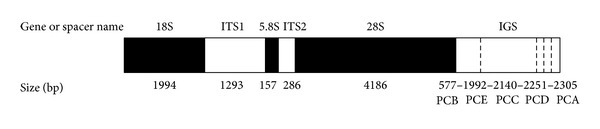
Organization of rRNA genes and spacers in* Paramphistomum cervi*. Dark shading indicates genes, light shading indicates spacers, and dashed bars in spacers indicate five lengths of IGS sequences.

All five complete rDNA sequences have been deposited in GenBank (accession numbers KJ459934–KJ459938). The lengths of five complete rDNA sequences were 8,493 bp, 9,908 bp, 10,056 bp, 10,167 bp, and 10,221 bp, respectively. The six regions (18S, ITS1, 5.8S, ITS2, 28S, and IGS) of the five complete rDNA sequences are shown in Figure 1.

### 3.2. 5.8S, ITS2, and 28S rDNA Analyses

There was no variation in the lengths of the 5.8S, ITS2, and 28S rDNA regions obtained from five* P. cervi* samples in this study, which were 157 bp, 286 bp, and 4,186 bp, respectively. The intraspecific variations within* P. cervi* were 0% for 5.8S and ITS2 and 0–0.5% for 28S rDNA. The G+C content was 54.14% for 5.8S, 51.75% for ITS2, and between 51.74% and 51.82% for 28S rDNA. The ITS2 rDNA sequences of the five samples and another stomach fluke from red deer (*Cervus elaphus*) in Slovakia [9] showed 100% identity. However, a comparative analysis revealed that the interspecific differences in ITS2 among members of the family Paramphistomatidae were 1.4–6.3%. Thus, the ITS2 sequence is a useful marker for taxonomic studies of the family Paramphistomatidae at the species level.

Despres et al. found no differences among the ITS2 sequences of* S. mansoni* from several geographical locations in Africa and the western hemisphere [25]. Similar results were reported for* E. revolutum*,* Clonorchis sinensis*, and* Opisthorchis viverrini* [26–28]. Thus, the ITS2 sequence is a useful marker for identifying closely related trematode species.

The present study reported the complete 5.8S and 28S rDNA sequences, which are the only representative sequences of the family Paramphistomatidae, of* P. cervi* for the first time. Therefore, no interspecific variations were investigated.

### 3.3. ITS1 rDNA Analyses

The length of the ITS1 rDNA sequences obtained from the five samples was 1,293 bp, which was longer than that of other trematodes, such as* O. felineus* and* C. sinensis* [28, 29]. The G+C content was 47.72–47.87%, which was lower than that of* C. sinensis* (54.2%) [28]. The intraspecific variations within* P. cervi* were 0–0.4%; thus, the nucleotide diversity in the ITS1 rDNA among the five samples was low. The result was in accordance with that of a study of* C. sinensis* [28].

**Figure 2:**
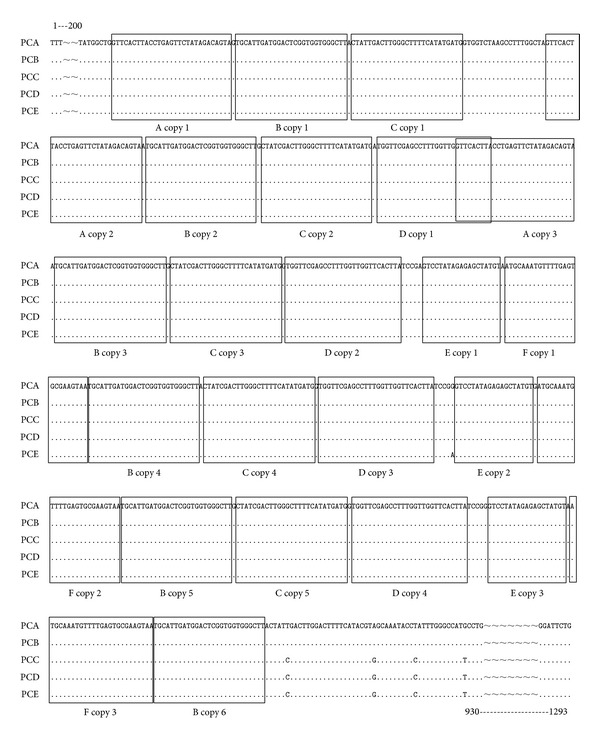
The alignment of ITS1 rDNA region of the five samples of* Paramphistomum cervi*. Dots denote sequence identity to the first sequence. Dashes represent nucleotide deletions.

It is of interest to note that ITS1 sequence in* P. cervi* contains repeated sequences with the following characteristics: three copies of a 23 nt complete direct repeat, A, located at 209 nt upstream of the ITS1 sequences; six copies of a 27 nt complete direct repeat, B, separated by 1 nt, which occur after repeat A; five copies of a 27 nt nearly complete direct repeat, C, separated by 1 nt, which occur after repeat B; four copies of a 28 nt complete direct repeat, D; three copies of a 19 nt direct repeat, E; and a 26 nt direct repeat, F (Figure 2).

Among trematodes, members of the genera* Dolichosaccus*,* Schistosoma*, and* Paragonimus* ITS1 have repeat sequences [30–33] similar to those found in this study. However, other species, such as* S. japonicum* and* Paragonimus westermani*, contain less repeats [32, 33]. As mentioned above, the length of the complete ITS1 sequence of* P. cervi* was longer than that of other trematodes, which is likely related to the number and organization of the repetitive elements.

Previous studies indicated that the ITS2 rDNA was more conserved than ITS1 [30], which may be because of the existence of diverse types and numbers of repeats. The long and short repeats leading to size variation were found across a range of helminthes, including trematodes [32–34], cestodes [35], and nematodes [36], but no length variation was detected in any of the* P. cervi* samples in the present study.

### 3.4. Analyses of IGS rDNA Sequences

The 5′ ends of the IGS from the* P. cervi* samples were determined by comparing them with previously published rDNA sequences of schistosomes [15, 37]. The 3′ terminus of the IGS was aligned readily, having relatively few indels and homologous regions in* S. japonicum* [15].

**Figure 3:**
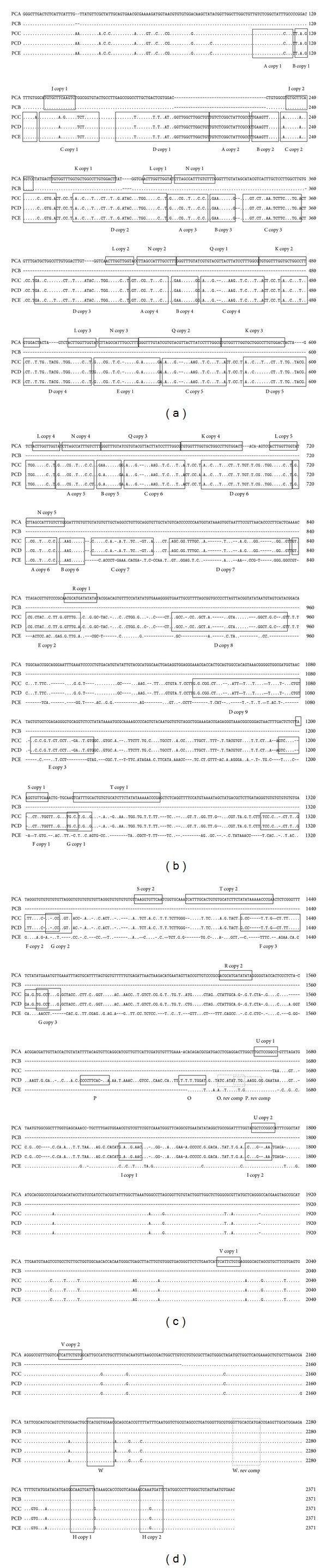
The alignment of intergenic spacer (IGS) rDNA sequences of the five individual* Paramphistomum cervi*. Dots denote sequence identity to the first sequence. Dashes represent nucleotide deletions.

The IGS rDNA of* P. cervi* had dynamic and highly complex structures. This became apparent upon amplification of the IGS rDNA's PCR products, which varied in length from 2,000 to 3,500 bp (not shown). After the removal of flanking 28S and 18S rDNA sequences, the lengths of the five IGS rDNA sequences were 577 bp, 1,992 bp, 2,140 bp, 2,251 bp, and 2,305 bp, respectively. The G+C content was 47.98–50.26%, and the pairwise sequence differences had a range of 0.2–46.3%. The IGS regions of the five* P. cervi* samples had strikingly different structures. In contrast to* S. intercalatum*,* S. haematobium*, and* S. japonicum*, the AT-rich regions of the five samples were absent, similar to the IGS region of* S. mansoni* [15, 37]. The IGS rDNAs of* P. cervi* could be roughly divided into two types based on their characteristics (Figure 3). The longest one (*P. cervi* sample 1, PCA) and the shortest one, PCB, were considered the same type; the other three samples had the second type. Only PCB contained one 13 nt complete direct repeat (J1 and J2) and one 12 nt incomplete inverted repeat (W and W reverse complement), which were missing the intervening sequences. PCA exhibited the following features: (1) 11 types (J, K, L, N, Q, R, S, T, U, V, and H), containing complete and incomplete direct repeats; (2) five types of short and incomplete inverted repeats (only W and W rep comp shown); and (3) a complete direct repeat H and an incomplete inverted repeat W (W rev comp) were shared by all five* P. cervi* samples. Compared with PCA and PCB, PCC-PCE had the following features: (1) nine types of complete and incomplete direct repeats (A, B, C, D, E, F, G, H, and I); (2) three types of inverted repeats W (W rev comp), O (O rev comp), and P (P rev comp); (3) incomplete inverted repeats O and P were only possessed by PCD; and (4) some differences were present in the intervening sequences of PCE. In contrast to the results of previous studies on* S. haematobium*,* S. intercalatum*,* S. mansoni*, and* S. japonicum* [15, 37],* P. cervi* was polytype. For example, it contained complete and incomplete direct repeats, as well as incomplete inverted repeats, and no identical direct or inverted repeat was found between the* Schistosoma* spp. and* P. cervi.*

Although the lengths and structures of the five IGS rDNA sequences of* P. cervi* were different from one another, some characteristics were similar. For example, the 5′ and 3′ termini of the five* P. cervi* samples' rDNA sequences were identical, indicating there were no length variations in this region. Similarly, there were no geographical or individual length variations in this region among samples of* S. japonicum* from several geographical locations in China [15].

Because no other IGS rDNA sequences of the family Paramphistomatidae were available in GenBank, the interspecific differences were not examined.

### 3.5. 18S rRNA Sequence Analysis and Reconstruction of Phylogenetic Relationships

The complete 18S rDNA sequence of* P. cervi* was determined by a comparison with those of* Paragonimus kellicotti* (HQ900670) and* E. revolutum* (GQ463130). The five 18S rDNA sequences obtained in this study were all 1,994 bp in length, and the G+C contents were 50.30–50.35%. A pairwise comparison of the aligned sequences was performed using MegAlign, and the comparison indicated that the intraspecific variations within* P. cervi* were between 0 and 0.2% for 18S.

**Figure 4:**
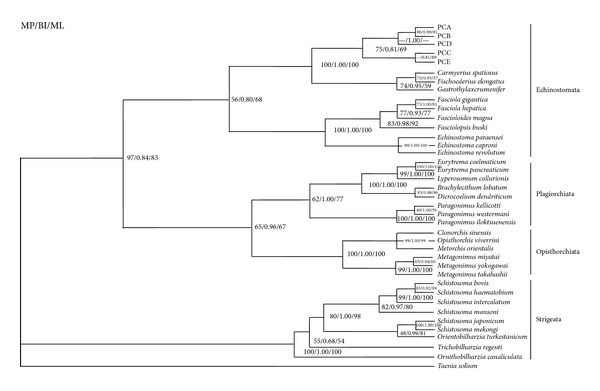
Inferred phylogenetic relationship among representative trematodes. The 18S rDNA sequences of* Paramphistomum cervi* were analyzed utilizing maximum parsimony (MP), Bayesian inference (BI), and maximum likelihood (ML), using* Taenia solium* (GenBank accession number GQ260091) as the outgroup. The numbers along branches indicate posterior probabilities and bootstrap values resulting from different analyses in the order: MP/BI/ML.

The 18S rRNA sequence is useful for studying the phylogeny of members of Digenea [38–40]. Using 18S rRNA sequences, the phylogenetic position of* P. cervi* was determined. Using MP, BI, and ML analyses, the phylogenetic relationships among members of trematodes were constructed based on sequences of the 18S rDNA sequences available in GenBank (Table 2) without gaps at both ends and with* Taenia solium* (GQ260091) as the outgroup. Three trees all placed* P. cervi* within the family Paramphistomatidae, as shown in Figure 4. Two main clades were observed. All the trematodes of Echinostomata, Plagiorchiata, and Opisthorchiata clustered together in one greater clade, and Strigeata clustered in another solitary clade, in accordance with morphological classifications. From the trees, the clade of Echinostomata was divided into two distinct clusters. The* P. cervi* (PCA-PCE) isolates,* Carmyerius spatiosus*,* Fischoederius elongatus*, and* Gastrothylax crumenifer* formed a tight cluster, while the Fasciolidae and Echinostomatidae occupied the proximate cluster as the sister group. These results indicated that the evolutionary relationship of* P. cervi* was closer to other members of the Paramphistomatidae than to other families (Fasciolidae, Echinostomatidae, Dicrocoeliidae, Paragonimidae, and Opisthorchiidae). The phylogenetic relationships of families within the Digenea were reported previously, indicating there were some discrepancies between the molecular features and some morphological characteristics [41], but this study was an exception.

In conclusion, the present study determined and characterized complete rDNA sequences from* P. cervi* samples for the first time. These results showed that the 5.8S and ITS2 rDNA sequences of* P. cervi* were quite conserved, with no within-species variation, but the IGS rDNA displayed the fastest evolutionary rate. These data provided novel and useful genetic markers for studying intra- and interspecific variation of the Paramphistomatidae and provided new sequence data for studying the systematics and phylogenetics of Digenea.

## Figures and Tables

**Table 1:** Primers used to amplify the complete rDNA sequence of *Paramphistomum cervi*. The upper and lower sequences are forward (F) and reverse (R) for each primer, respectively.

Name of primer	Amplification regions	Primer sequence (5′-3′)	Annealing temperature (°C)	Length
P1	18S	F: TCTGTGATGACTCTGGAT	53.7	1,596 bp
		R: ACCATTCAATCGGTAGTA		
P2	18S-28S	F: CACCGCCCGTCGCTACTACC	55.2	1,303 bp
		R: TACTTTTCAACTTTCCCTCA		
P3	28S-1	F: TAGGCAATGTGGTGTT	54.7	1,156 bp
		R: TTGCACGTCAGAATCGCT		
P4	28S-2	F: CGGAGACGGCGGCTTGTTGTG	57.1	1,608 bp
		R: GGCTGTTCACCTTGGAGA		
P5	28S-3	F: ACAGAGACGGGGTGCCTG	51.7	1,390 bp
		R: AAAATCAAAATCAAGTAA		
P6	28S-18S (IGS)	F: TACCACCACCGTCATTGTTTCTTTG	55.7	1,742 bp
		R: AAGTTATCCAGAGTCATCACAGAGT		

**Table 2:** Sequences of 18S rDNA available in GenBank used to construct phylogenetic relationships among the trematodes.

Species	GenBank accession number	Length (bp)	Classification
*Eurytrema pancreaticum *	DQ401034	1,857	Plagiorchiata
*Eurytrema coelmaticum *	DQ401035	1,857	Plagiorchiata
*Lyperosomum collurionis *	AY222143	1,945	Plagiorchiata
*Brachylecithum lobatum *	AY222144	1,945	Plagiorchiata
*Dicrocoelium dendriticum *	Y11236	1,950	Plagiorchiata
*Paragonimus westermani *	AJ287556	1,902	Plagiorchiata
*Paragonimus kellicotti *	HQ900670	1,870	Plagiorchiata
*Paragonimus iloktsuenensis *	AY222141	1,860	Plagiorchiata
*Metorchis orientalis *	JF314771	1,901	Opisthorchiata
*Opisthorchis viverrini *	JF823987	1,889	Opisthorchiata
*Clonorchis sinensis *	JF314770	1,902	Opisthorchiata
*Metagonimus yokogawai *	HQ832632	1,867	Opisthorchiata
*Metagonimus miyatai *	HQ832626	1,867	Opisthorchiata
*Metagonimus takahashii *	HQ832629	1,867	Opisthorchiata
*Fasciola hepatica *	AJ004969	1,941	Echinostomata
*Fasciola gigantica *	AJ011942	1,945	Echinostomata
*Fascioloides magna *	EF534989	1,934	Echinostomata
*Fasciolopsis buski *	L06668	1,978	Echinostomata
*Echinostoma revolutum *	AY222132	1,871	Echinostomata
*Echinostoma caproni *	L06567	1,977	Echinostomata
*Echinostoma paraensei *	FJ380226	1,836	Echinostomata
*Carmyerius spatiosus *	JX518972	1,858	Echinostomata
*Fischoederius elongatus *	JX518979	1,859	Echinostomata
*Gastrothylax crumenifer *	JX518984	1,858	Echinostomata
*Schistosoma mekongi *	AY157228	1,880	Strigeata
*Schistosoma japonicum *	AY157226	1,883	Strigeata
*Schistosoma haematobium *	Z11976	1,972	Strigeata
*Schistosoma intercalatum *	AY157235	1,863	Strigeata
*Schistosoma bovis *	AY157238	1,864	Strigeata
*Schistosoma mansoni *	U65657	1,989	Strigeata
*Orientobilharzia turkestanicum *	AF442499	1,909	Strigeata
*Trichobilharzia regenti *	AY157218	1,872	Strigeata
*Ornithobilharzia canaliculata *	AY157222	1,866	Strigeata
*Taenia solium* (outgroup)	GQ260091	2,599	
